# Factors impacting the efficacy of the in-situ vaccine with CpG and OX40 agonist

**DOI:** 10.1007/s00262-023-03433-3

**Published:** 2023-04-05

**Authors:** Alexander A. Pieper, Dan V. Spiegelman, Mildred A. R. Felder, Arika S. Feils, Noah W. Tsarovsky, Jen Zaborek, Zachary S. Morris, Amy K. Erbe, Alexander L. Rakhmilevich, Paul M. Sondel

**Affiliations:** 1 Department of Human Oncology, School of Medicine and Public Health, University of Wisconsin, Madison, WI USA; 2 Department of Biostatistics and Medical Informatics, School of Medicine and Public Health, University of Wisconsin, Madison, WI USA; 3 Department of Pediatrics, School of Medicine and Public Health, University of Wisconsin, Madison, WI USA; 4 4159 MACC Fund UW Childhood Cancer Research Wing, Wisconsin Institute for Medical Research, University of Wisconsin, 1111 Highland Avenue, Madison, WI 53705-2275 USA

**Keywords:** In-situ vaccine, CpG, OX40 agonist, Anti-CTLA-4, Preclinical tumor progression

## Abstract

**Background:**

The *in-situ* vaccine using CpG oligodeoxynucleotide combined with OX40 agonist antibody (CpG + OX40) has been shown to be an effective therapy activating an anti-tumor T cell response in certain settings. The roles of tumor volume, tumor model, and the addition of checkpoint blockade in the efficacy of CpG + OX40 *in-situ* vaccination remains unknown.

**Methods:**

Mice bearing flank tumors (B78 melanoma or A20 lymphoma) were treated with combinations of CpG, OX40, and anti-CTLA-4. Tumor growth and survival were monitored. In vivo T cell depletion, tumor cell phenotype, and tumor infiltrating lymphocyte (TIL) studies were performed. Tumor cell sensitivity to CpG and macrophages were evaluated in vitro.

**Results:**

As tumor volumes increased in the B78 (one-tumor) and A20 (one-tumor or two-tumor) models, the anti-tumor efficacy of the *in-situ* vaccine decreased. In vitro, CpG had a direct effect on A20 proliferation and phenotype and an indirect effect on B78 proliferation via macrophage activation. As A20 tumors progressed in vivo, tumor cell phenotype changed, and T cells became more involved in the local CpG + OX40 mediated anti-tumor response. In mice with larger tumors that were poorly responsive to CpG + OX40, the addition of anti-CTLA-4 enhanced the anti-tumor efficacy in the A20 but not B78 models.

**Conclusions:**

Increased tumor volume negatively impacts the anti-tumor capability of CpG + OX40 *in-situ* vaccine. The addition of checkpoint blockade augmented the efficacy of CpG + OX40 in the A20 but not B78 model. These results highlight the importance of considering multiple preclinical model conditions when assessing the efficacy of cancer immunotherapy regimens and their translation to clinical testing.

**Supplementary Information:**

The online version contains supplementary material available at 10.1007/s00262-023-03433-3.

## Introduction

Emerging clinical evidence indicates patients with greater tumor burden have reduced responses to immune checkpoint blockade (ICB) [1–3]. Some preclinical data demonstrate that larger tumor burdens are more immunosuppressive at baseline [4–6], and these baseline characteristics contribute to larger tumors being less responsive to ICB and other immunotherapy strategies [4, 7–9]. However, other reports demonstrate that anti-tumor efficacy of immunotherapy either did not correlate with [10] or improved with increased tumor burden [11]. We have previously published the effects of immunocytokines (ICs, tumor antigen specific antibodies linked to stimulatory cytokines) as *in-situ* vaccines in various preclinical models [7, 12–14]. In the NXS2 neuroblastoma model [7], intratumoral (IT) injections of a GD2 targeted IC significantly improved survival over control mice if tumors were less than 34 mm^3^ but not if tumor volumes were greater than 34 mm^3^. The relationship between tumor volume, efficacy, and mechanism of *in-situ* vaccines has not been fully characterized.

The combination of CpG oligodeoxynucleotide and OX40 agonist antibody (CpG + OX40) can activate a potent *in-situ* vaccine effect in the preclinical A20 B-cell lymphoma model [15–17]. IT injections of CpG + OX40 can cure mice of both a treated tumor and distant untreated tumor in a T cell dependent manner [17]. CpG is a pathogen associated molecular pattern (PAMP) molecule that activates toll like receptor 9 (TLR-9) in macrophages, dendritic cells, and natural killer cells [18]. The OX40 molecule is a costimulatory receptor expressed by T cells after T cell receptor (TCR) engagement [19]. When activated, OX40 provides a “second signal” for T cell activation [19–21]. OX40 activation on T regulatory cells (Tregs) can lead to Treg apoptosis, decreased production of interleukin (IL)-10, and decreased FoxP3 expression [22–24]. In the A20 model, we and others have shown IT injections of CpG can increase OX40 expression on CD4 + effector and CD8 + T cells in the tumor microenvironment (TME) [17, 25].

In this report, we tested how tumor volume and tumor model influenced the anti-tumor efficacy and mechanism of the *in-situ* vaccine CpG + OX40 in two preclinical tumor types where clinical IT injections are clinically achievable due to tumor accessibility. As tumors progress, CpG + OX40 lost effectiveness in the B78 melanoma and A20 lymphoma models. In vitro macrophage co-culture experiments suggested that CpG is functioning differently in the B78 and A20 models to slow tumor progression. As untreated A20 tumors became larger in vivo, tumor phenotypic changes occurred, which correlated with the blunted effect of the T cell mediated *in-situ* vaccine. T cell depletion studies in mice bearing two separate A20 tumors demonstrated different T cell requirements for a local anti-tumor response to CpG + OX40 depending on the starting volumes of the two tumors. Finally, in conditions that were unresponsive to CpG + OX40 alone, the addition of anti-CTLA-4 restored the tumor curing ability of CpG + OX40 in A20-bearing mice, but not B78-bearing mice.

## Methods

### Mice

Female *C57BL/6* and *Balb/c* 6–8 week old mice were purchased from Taconic Biosciences (Rensselaer, NY) and housed in accordance with the Guide for Care and Use of Laboratory Mice. Experiments were performed under an institutional animal care and use committee approved animal protocol in accordance with NIH guidelines. Per our institutionally approved protocol, mice were euthanized when any tumor size reached 20 mm in the longest dimension or when they showed substantial systemic effects of the enlarging tumor.

### Cell culture

A20 lymphoma was obtained from Dr. Stephen Gillies in 2017. To achieve consistent tumor engraftment, A20 cells were passaged in vivo as previously described [25]. B78-D14 (B78) melanoma was obtained from Dr. Ralph Reisfeld in 2002. B78 and A20 cells were cultured as previously described [25].

### In vivo tumor models

A20 (5 × 10^6^) and B78 (2 × 10^6^) cells were injected intradermally into the right and/or left flank of *Balb/c* mice (A20) and *C57BL/6* mice (B78). Tumors were measured twice per week, and tumor volumes were calculated as previously described [14]. Once tumor volumes reached target size, mice were randomized to have similar average starting tumor volumes for the tumor scheduled to receive IT treatment. Tumor volumes were categorized as follows: first-palpable (~ 15 mm^3^), small (~ 100 mm^3^), moderate (~ 200 mm^3^), large (~ 350 mm^3^), and advanced (~ 1000 mm^3^). B78 tumor volumes reached first palpable (~ 15 mm^3^) and small (~ 100 mm^3^) sizes 10 days and 21 days after tumor cell injection. A20 tumor volumes reached small (~ 100 mm^3^), moderate (~ 200 mm^3^), large (~ 350 mm^3^), and advanced (~ 1000 mm^3^) sizes 8, 14, 18, and 24 days after tumor cell injections. Mice were randomized at these time points (± 1 day) after tumor cell injections for the given experiments*.* Mice were euthanized when the greatest tumor dimension reached 20 mm or, specifically in the A20 model, the mouse became moribund due to metastatic disease. A complete response (CR) was defined as complete resolution of all detectable tumor(s). In mice with 2 tumors, a local complete response (LCR) was defined as complete resolution of tumor at the local site (either the treated or untreated tumor). Cure is defined as complete resolution of all tumor(s) in an individual animal that was maintained, without any sign of recurrent tumor, until the end of the monitoring period (normally 90 days).

### Immunotherapy and antibodies

CpG (50 μg; 1826 from Integrated DNA Technologies) and anti-OX40 (20 μg; OX86 clone, European Collection of Cell Cultures, harvested and isolated from immunodeficient mice [26]) in 60μL PBS were injected IT with a 29½ gauge insulin syringe on days 0, 2, and 4 after randomization. Anti-CTLA-4 (200 μg in 100μL PBS; clone 9D9 from Bristol Myers Squibb) was injected intraperitoneally (IP) with a 26 gauge needle on days 2, 5, and 8. Anti-CD4 (400 μg; clone GK1.5 from Bio X Cell) and anti-CD8 (400 μg; clone 2.43 from Bio X Cell) in 200μL PBS were injected IP on days − 1, 6, and 13 for T cell depletion experiments [27].

The antibodies used for flow cytometry analysis are listed in Table S1.

### Tumor cell and tumor infiltrating immune cell analysis

B78 tumors were grown as described above. A20 tumors were grown by injecting 5 × 10^6^ (large) or 1 × 10^6^ (small) A20 cells. Once tumors reached target volumes, mice were randomized, treated, and tumors were harvested and prepped as described [25, 27]. Brilliant stain buffer (BD Biosciences) and the FoxP3/Transcription Factor Staining Buffer Set (eBioscience) were used per manufacturer instructions. TLR-9 expression on cultured tumor cells was determined as described [28].

All data were collected on an Attune flow cytometer (ThermoFisher) and analyzed with FlowJo v10 software (BD). Tregs were defined as CD45 + /CD3 + /CD4 + /CD25 + /FoxP3 + ; CD4 + cells not CD25 + and FoxP3 + double positive were defined as non-regulatory CD4 + T cells.

### Macrophage anti-tumor effect in vitro

Murine peritoneal exudate cells were obtained by peritoneal lavage and plastic adhesion, as described [28]. Adhesion-purified macrophages from *C57BL/6* or *Balb/c* mice were incubated with B78 or A20 tumor cells (1 × 10^4^ per well) for 48 h in medium with or without CpG (5 or 50* µg*/ml), and a [^3^H]thymidine incorporation assay was performed [28]. Results are expressed as counts per 5 min for triplicate wells ± standard error of the mean (SEM). Macrophages alone (in the absence of tumor cells or CpG) incorporated negligible amounts of [^3^H]thymidine [28].

### Statistical analysis

Average group tumor volumes are plotted showing mean ± SEM. Tumor volume plots were summarized by time-weighted average (area under the volume-time curve, calculated using trapezoidal method). Time-weighted averages were compared between treatment groups overall by Kruskal–Wallis tests. If significant by Kruskal–Wallis test, pairwise comparisons were conducted using Mann–Whitney tests. Survival data were plotted using Kaplan–Meier methods and analyzed using log-rank comparisons. Flow cytometry results are plotted as mean ± SEM, with each symbol representing results from one mouse/sample. Flow cytometry data were analyzed via Mann–Whitney tests (when comparing two groups) or one-way ANOVA with Tukey’s multiple comparison corrections (when comparing three or more groups). Complete response rates were analyzed overall with chi-square tests (when comparing three or more groups). If significant by chi-square tests, pairwise comparisons were conducted using two-sample tests of proportions. [^3^H]thymidine results are plotted as mean ± SEM fold change differences comparing wells of a particular experiment and macrophage condition to the intra-experimental media average. Experimental fold change differences were analyzed via two-way ANOVA with Tukey’s multiple comparison corrections. *P* values < 0.05 were considered significant and were indicated in all figures as follows: *P ≤ 0.05; **P ≤ 0.01; ***P ≤ 0.001; ****P ≤ 0.0001; ns—nonsignificant. Each tumor volume experiment shown was repeated at least once.

## Results

### CpG + OX40 cures mice of first-palpable B78 melanomas but fails to control growth of small B78 melanomas

**Fig. 1:**
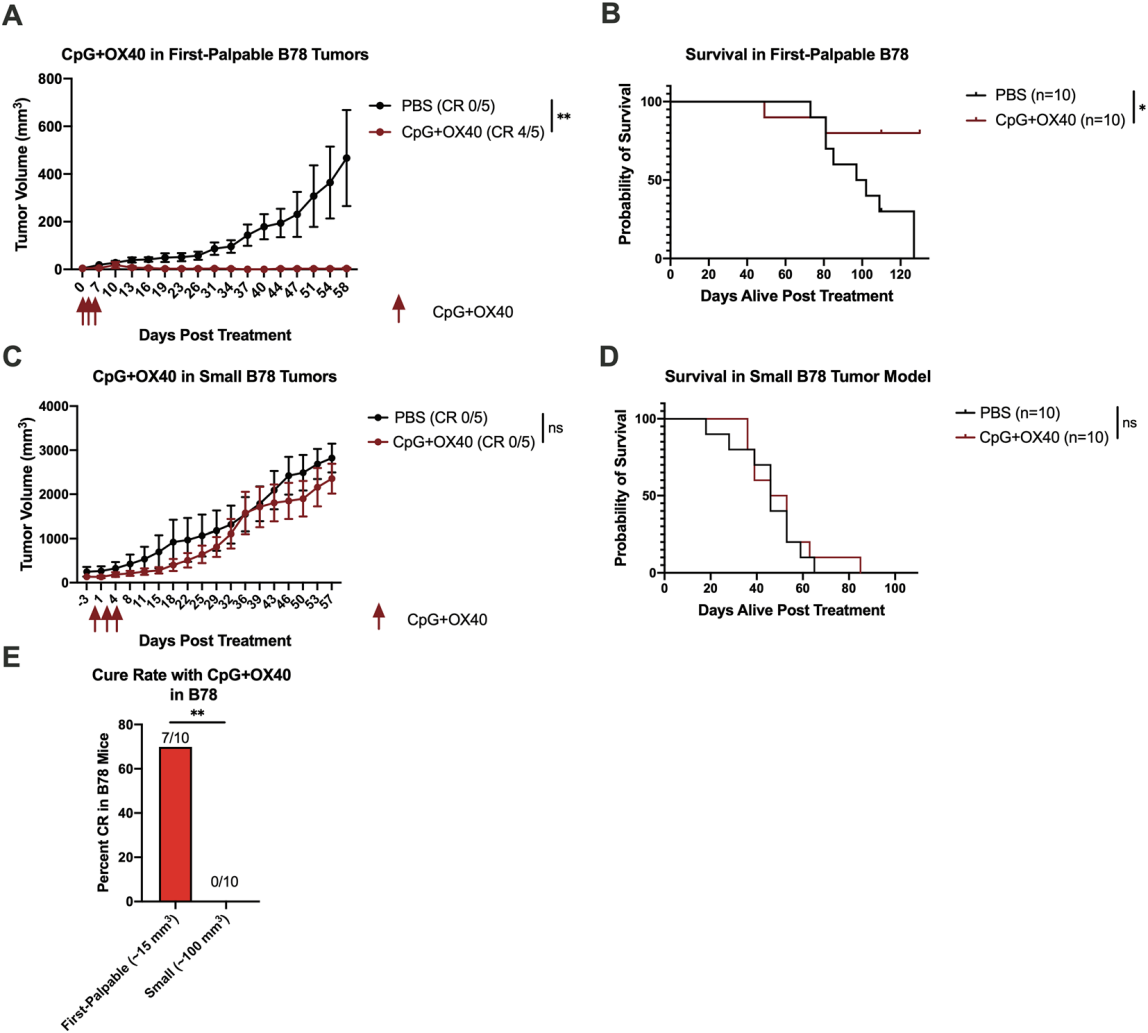
CpG + OX40 *in-situ* vaccine cures mice of first-palpable (~ 15 mm^3^) B78 tumors but fails to slow tumor progression of small (~ 100 mm^3^) B78 Tumors. **a** Average tumor volume (± SEM) from a representative experiment and **b** combined overall survival from two independent experiments showing responses to PBS (black) or CpG + OX40 (red) in the first-palpable (~ 15 mm^3^) B78 tumor model. **c** Average tumor volume (± SEM) from a representative experiment and **d** the combined overall survival from two independent experiments showing responses to PBS (black) or CpG + OX40 (red) in the small (~ 100 mm^3^) B78 model. The number of mice demonstrating a complete response (CR) in **a** and **c** is shown in parentheses. **e** The percent of mice bearing first-palpable or small B78 tumors that were cured with CpG + OX40 treatment. *P* values for tumor volume plots were calculated using time-weighted average analysis. *P* values for overall survival were calculated via log rank test. *P* value for cure rate calculated via chi-square test. *P ≤ 0.05; **P ≤ 0.01; ns, not significant

We initially tested the anti-tumor capability of CpG + OX40 in the immunologically cold B78 model when tumors were first-palpable (~ 15 mm^3^). At this size, CpG + OX40 demonstrated a significant anti-tumor effect, curing 7 of 10 mice, and significantly improving survival over control mice (Fig. 1a, b, e). Next, we tested the anti-tumor efficacy of CpG + OX40 in small (~ 100 mm^3^) B78 tumors. Unlike ~ 15 mm^3^ B78 tumors, ~ 100 mm^3^ B78 tumors showed no significant anti-tumor response to CpG + OX40 (Fig. 1c). The survival benefit provided by CpG + OX40 in first-palpable B78 tumors was lost in the small B78 model (Fig. 1d), and no mice were cured of their tumor (Fig. 1e).

Flow cytometry analyses were performed on untreated small (~ 100 mm^3^) and large (~ 350 mm^3^) B78 tumors to explore if there were baseline immunologic or tumor phenotype differences as B78 tumors progress in size. No significant baseline TIL or tumor phenotype differences were measured (Fig. S1A–C). Significantly fewer live tumor cells (GD2 + cells) per total events were counted in large tumors compared to small tumors, suggesting increased levels of tumor necrosis as B78 tumors progress (Fig. S1D). In separate flow experiments, CpG + OX40 treatment significantly lowered the Treg/CD45 + ratio in the TME of small B78 tumors (Fig. S1E).

### CpG + OX40 cures mice of moderate and large A20 lymphoma tumors but fails to cure in the advanced disease setting

**Fig. 2:**
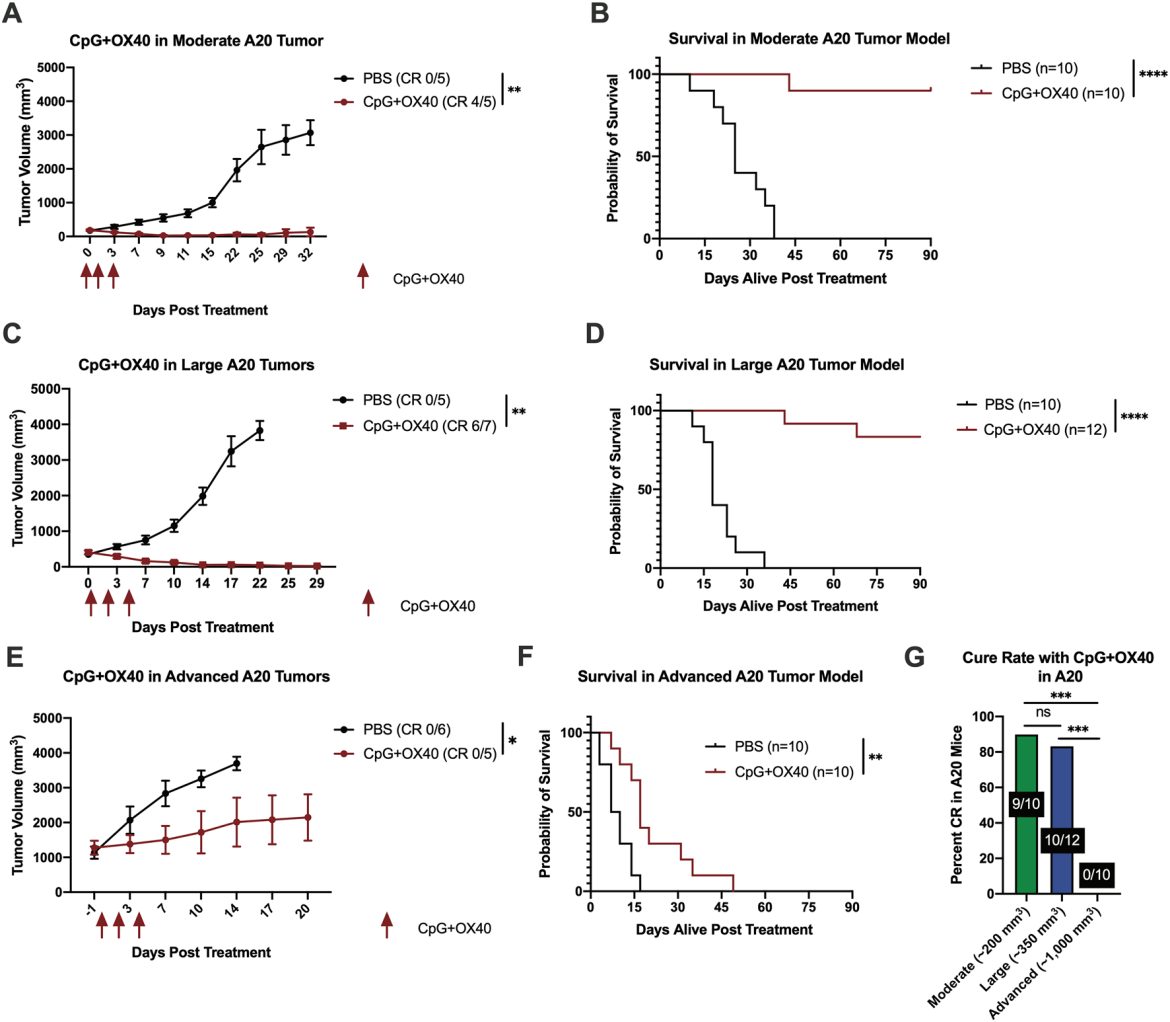
Decreased Curative Effect of CpG + OX40 *In-situ* Vaccine in A20 Model as Tumor Burden Increases. **a, c, e** Average tumor volume (± SEM) from a representative experiment and **b, d, f** combined overall survival from two independent experiments of mice bearing a **a, b** moderate (~ 200 mm^3^), **c, d** large (~ 350 mm^3^), **e, f** or advanced (~ 1000 mm^3^) A20 flank tumor treated with PBS (black) or CpG + OX40 (red). The number of mice cured (CR), based on showing a complete response and remaining tumor-free to day 90, of their tumor in **a**, **c**, and **e** is shown in parentheses. **g** The percent of mice bearing a single moderate (green), large (blue), or advanced (purple) A20 tumor that were cured with CpG + OX40 treatment. *P* values for tumor volume plots were calculated using time-weighted average analysis. *P* values for overall survival were calculated via log rank test. P value for cure rate calculated via chi-square tests. *P ≤ 0.05; **P ≤ 0.01; ***P ≤ 0.001; ****P ≤ 0.0001; ns, not significant

We next demonstrated the effect of tumor size on response to CpG + OX40 in *Balb/c* mice bearing a single A20 tumor. In separate experiments, we compared PBS vs. CpG + OX40 in mice bearing moderate (~ 200 mm^3^), large (~ 350 mm^3^), or advanced (~ 1000 mm^3^) A20 tumors. In mice with moderate or large A20 tumors, CpG + OX40 activated a strong anti-tumor response, causing significant tumor regression, improving survival over control, and most mice were cured of their tumor (Fig. 2a–d, g). In contrast, advanced (~ 1000 mm^3^) A20 tumors treated with CpG + OX40 continued to grow progressively, though tumor growth was significantly slowed (Fig. 2e). As a result, all treated mice died (Fig. 2f). Thus, the curative potential of CpG + OX40 seen in the moderate and large A20 models was lost in the advanced model (Fig. 2g).

### A20 tumor phenotype changes with size progression

**Fig. 3:**
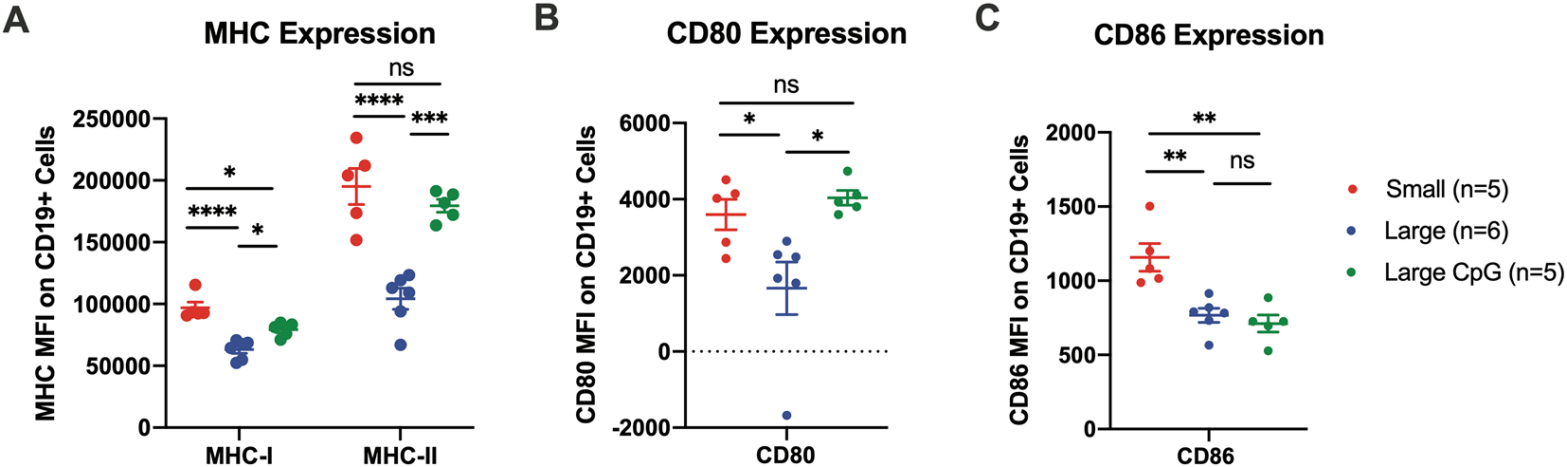
In Vivo Tumor Phenotypic Changes with Tumor Progression and Intratumoral CpG. Average (± SEM) median fluorescent intensity (MFI) of **a** MHC-I and MHC-II, **b** CD80, and **c** CD86 on CD19 + cells (e.g. A20 tumor cells) in small (~ 150 mm^3^) untreated (red), large (500 mm^3^) untreated (blue), and large (500 mm^3^) CpG treated (green) A20 tumors. Large CpG treated tumors were injected once, intratumorally, with 50 μg of CpG and all three tumor groups were harvested together, 48 h after CpG injection. P values calculated via one-way ANOVA analysis with Tukey’s multiple comparison correction. *P ≤ 0.05; **P ≤ 0.01; ***P ≤ 0.001; ****P ≤ 0.0001; ns, not significant

To evaluate tumor phenotype changes as A20 tumors progress in size, we harvested untreated small (~ 150 mm^3^) and large (~ 500 mm^3^) A20 tumors for flow analysis (gating strategy presented in Fig. S2). CD19 + cells in large tumors demonstrated significantly lower MHC–I, MHC-II, CD80, and CD86 expression compared to small tumors (Fig. 3a–c). TIL analysis on untreated small and large A20 tumors demonstrated trends toward increased ratios of CD3 + /CD19 + , CD4 + non-Treg/CD19 + , and CD8 + /CD19 + (*p* = 0.0519, 0.126, 0.126, respectively) in small tumors compared to large tumors (Fig. S3). There was a small but significant increase in the Treg/CD19 + ratio in small tumors compared to large tumors (Fig. S3).

### CpG acts differently on B78 and A20 tumors: indirectly preventing B78 proliferation via macrophages, while acting directly to slow A20 cell proliferation

We were interested in what effect CpG and CpG-activated *C57BL/6* or *Balb/c* peritoneal derived macrophages had on B78 and A20 proliferation. In the absence of macrophages, CpG appeared to increase B78 proliferation though B78 cells do not express TLR-9 (Fig. S4A, B). *C57BL/6* derived macrophages significantly slowed B78 proliferation in a dose dependent manner when activated with CpG (Fig. S4A).

Taken together, the above data suggest CpG + OX40 is functioning, in part, by activating tumor associated macrophages (Fig. S4A) and decreasing the frequency of tumor infiltrating Tregs in the B78 model (Fig. S1E). If B78 tumors are treated early, these immunologic modifications likely contribute to curing mice of their tumor burden. However if B78 tumors are allowed to progress (~ 100 mm^3^), the activation of macrophages and decrease of Tregs is insufficient to slow tumor progression or prolong survival.

Others have published that CpG increases immune co-stimulatory molecule expression on mouse and human B cell lymphomas in vitro [29, 30]. We observed similar in vivo phenotypic changes on CD19 + cells 48 h after IT injection of CpG. CD19 + cells from large (~ 500 mm^3^) A20 tumors treated with CpG demonstrated significantly increased MHC-I, MHC-II, and CD80 expression compared to large untreated tumors (Fig. 3a, b). These phenotypic changes following CpG exposure were replicated in vitro (Fig. S4E), suggesting that CpG is acting directly on A20 cells via their confirmed TLR-9 (Fig. S4D) to modify tumor cell phenotype.

To compare the CpG effects on A20 cells to B78 cells, a macrophage co-culture experiment was conducted with macrophages harvested from *Balb/c* mice. Unlike B78 cells (Fig. S4A), A20 tumor cells demonstrated significantly decreased proliferation when cultured in the presence of CpG (Fig. S4C). Adding macrophages, either *Balb/c* derived (Fig. S4C) or *C57BL/6* (not shown), to the culture did not further decrease proliferation. These in vitro and in vivo studies indicate CpG is functioning differently in the B78 and A20 models.

### Systemic anti-tumor effect of CpG + OX40 decreases with increasing systemic A20 tumor burden

**Fig. 4:**
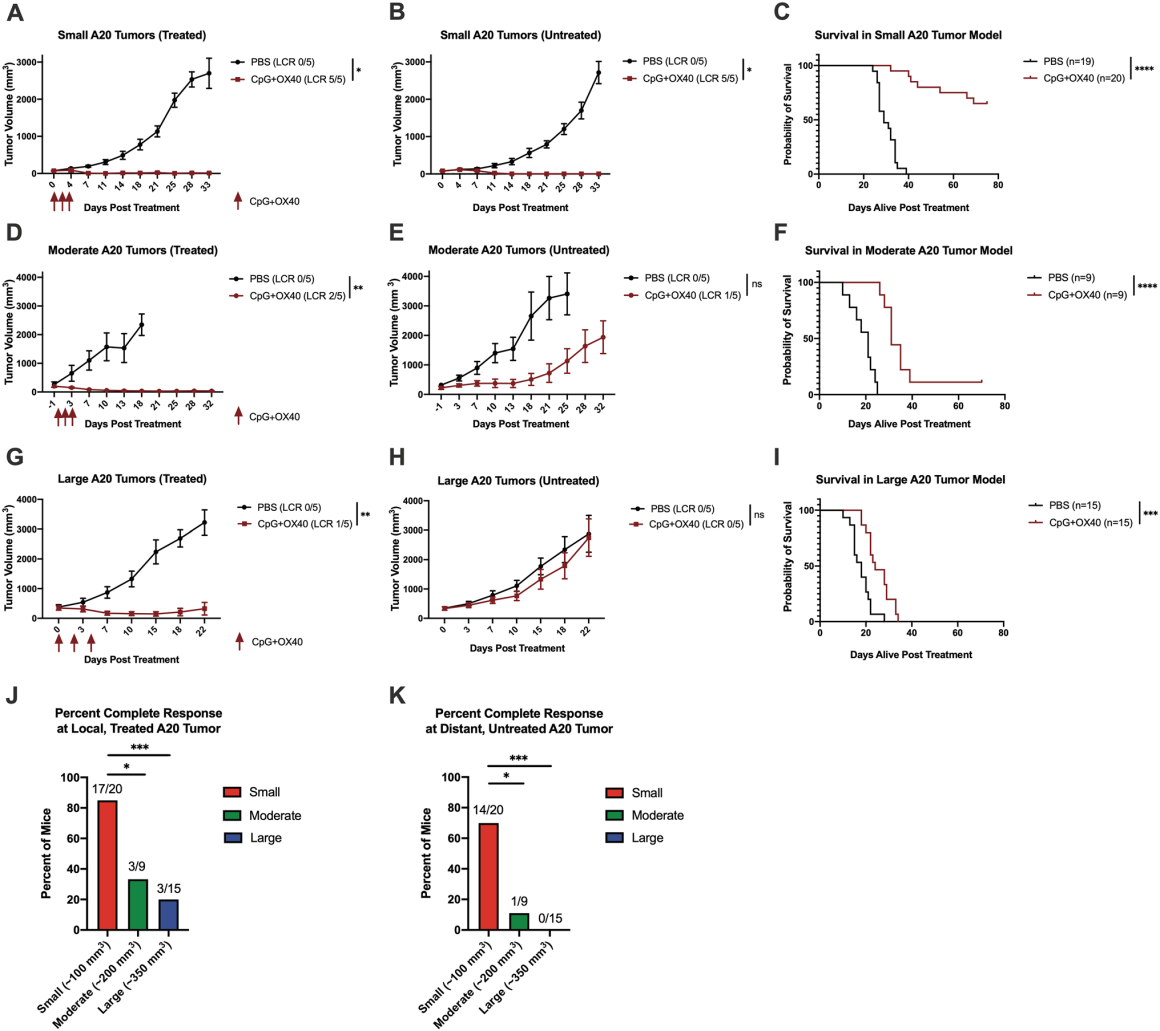
Systemic in-situ vaccination strength decreases with increased systemic A20 tumor burden*. Balb/c* mice bearing 2 separate A20 tumors [**(a–c)** small (~ 100 mm^3^), **(d–f)** moderate (~ 200 mm^3^), and **(g–i)** large (~ 350 mm^3^) were treated with PBS (black) or CpG + OX40 (red). Average tumor volume (± SEM) are shown for the treated tumor **(a, d, g)**, and the distant untreated tumor **(b, e, h)**. The combined overall survival for each group is shown **(c, f, i)**, using data pooled from at least 2 experiments. The number of mice demonstrating a local complete response (LCR) at the treated tumor or LCR specifically at the distant untreated tumor are shown in parentheses in **(a, d, g)** and **(b, e, h)**, respectively. The percent of mice from combined experiments bearing small (red), moderate (green), or large (blue) tumors that demonstrated a LCR to CpG + OX40 at the **(j)** local, treated tumor and **(k)** distant, untreated tumor. P values for tumor volume plots were calculated using time-weighted average analysis. *P* values for overall survival were calculated via log rank test. *P* values for complete response data calculated via chi-square tests. *P ≤ 0.05; **P ≤ 0.01; ***P ≤ 0.001; ****P ≤ 0.0001; ns, not significant. All comparisons that are not shown with a *, **, *** or **** are not significantly different from each other

Using three separate two-tumor A20 models that varied in tumor volume, we investigated the strength of the systemic anti-tumor response of CpG + OX40 in the setting of increased systemic tumor burden. In one experiment in this model (~ 100 mm^3^ per tumor), CpG alone and OX40 alone were tested to validate the results others have published with these drugs in this model (Fig. S5). Similar to what others have described [17], CpG alone had a strong local anti-tumor effect but failed to slow distant, untreated tumor progression. OX40 alone slowed local and distant tumor progression, but CpG + OX40 demonstrated the strongest anti-tumor effect at both the local and distant tumors (Fig. S5). In the small two-tumor model (~ 100 mm^3^ per tumor), CpG + OX40 had a significant anti-tumor effect at both the treated and untreated tumors (Fig. 4a, b). CpG + OX40 significantly prolonged survival over PBS, and 70% of mice were cured (Fig. 4c, j, k).

In the moderate (~ 200 mm^3^ per tumor) two-tumor model, CpG + OX40 was effective at causing local tumor regression (Fig. 4d); however, after controlling distant tumor progression early in the experiment (days 3–18), tumor growth increased rapidly after day 18 (Fig. 4e). CpG + OX40 significantly prolonged survival compared to PBS in this moderate sized (~ 200 mm^3^) two-tumor model (Fig. 4f); however, 8 of 9 mice died of tumor progression before day 40.

In the large two-tumor model (~ 350 mm^3^ per tumor), the local tumor curing effects of CpG + OX40 observed with small tumors were significantly decreased (Fig. 4g, j), and there was no significant slowing of distant tumor progression (Fig. 4h). CpG + OX40 did maintain a small but significant prolongation of survival over PBS in this model (Fig. 4i), but no mice were cured with CpG + OX40 (Fig. 4j–k). As tumor burden increased, CpG + OX40 induced a significantly smaller percentage of local complete responses at the treated and untreated tumors (Fig. 4j–k).

### Differing requirements of T cells for local and systemic anti-tumor response in small and large two-tumor A20 models

Given the varying effects of CpG + OX40 in the small and large two-tumor models (Fig. 4), we next tested the requirement of T cells following CpG + OX40 in T cell depletion studies.

**Fig. 5:**
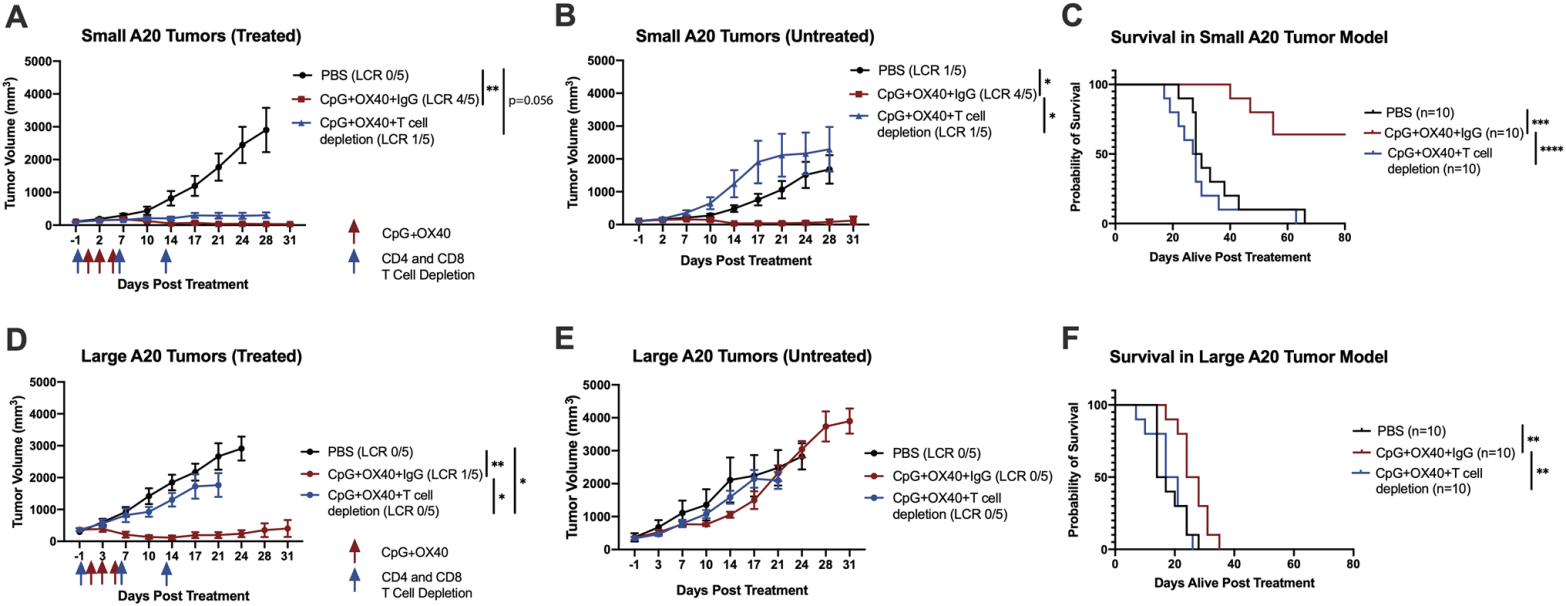
Differing requirements of T cells for local anti-tumor response in small and large two-tumor A20 models. Representative average tumor volume (± SEM) of the **a, d** local, treated tumor, **b, e** distant, untreated tumor, and **c, f** combined overall survival from two independent experiments of mice treated with PBS (black), CpG + OX40 + Rat IgG (red), or CpG + OX40 + CD4 depletion + CD8 depletion (blue) in the **(a–c)** small (~ 100 mm^3^) and **(d–f)** large (~ 350 mm^3^) two-tumor A20 models. The number of mice demonstrating a local complete response (LCR) at the treated tumor or distant untreated tumor are shown in parentheses in **(a, d)** and **(b, e**), respectively. *P* values for tumor volume plots were calculated using time-weighted average analysis. P values for overall survival were calculated via log rank test. *P ≤ 0.05; **P ≤ 0.01; ***P ≤ 0.001; ****P ≤ 0.0001. All comparisons that are not shown with an *, **, *** or **** are not significantly different from each other

In the small two-tumor model (~ 100 mm^3^), depleting CD4 + and CD8 + T cells did not prevent CpG + OX40 from significantly inhibiting local tumor progression (Fig. 5a). In the absence of T cells, CpG + OX40 lost its ability to control distant, untreated tumor progression (Fig. 5b), and the cohort of mice receiving T cell depletion were euthanized at a similar rate as the PBS control (Fig. 5c).

In the large two-tumor model (~ 100 mm^3^), mice in the CpG + OX40 and T cell depletion cohort demonstrated local tumor progression (Fig. 5d). At the distant, untreated tumors, all cohorts demonstrated a similar lack of anti-tumor effect (Fig. 5e). Although the treatment with CpG + OX40 and IgG significantly prolonged survival (Fig. 5f), the effect was weaker than for the small tumor model (Fig. 5c), with all treated animals dying before day 40.

### Effects of systemic anti-CTLA-4 to CpG + OX40 in poorly responsive tumor models

After establishing that the CpG + OX40-induced distant anti-tumor effect was T cell dependent (Fig. 5b) and CD19 + cells in large A20 tumors express decreased CD80 and CD86 (Fig. 3), we hypothesized the addition of anti-CTLA-4 would augment T cell activation and enhance the anti-tumor effect of CpG + OX40 in the poorly responsive, large two-tumor A20 model given that CD80/86 are involved in T cell activation through the CD28/CTLA-4 axis [31]. Furthermore, we have previously shown additional Treg depletion via anti-CTLA-4 (IgG2a isotype) enables augmented cure rates in mice bearing larger B78 tumors that are no longer responding to a separate *in-situ* vaccine [radiation therapy (RT) + IT-IC] [14, 32]. Thus, we randomized mice bearing either a poorly responsive small (~ 100 mm^3^) B78 tumor or two large (~ 350 mm^3^ per tumor) A20 tumors into 4 treatment groups: PBS, anti-CTLA-4, CpG + OX40, and CpG + OX40 + anti-CTLA-4.

**Fig. 6:**
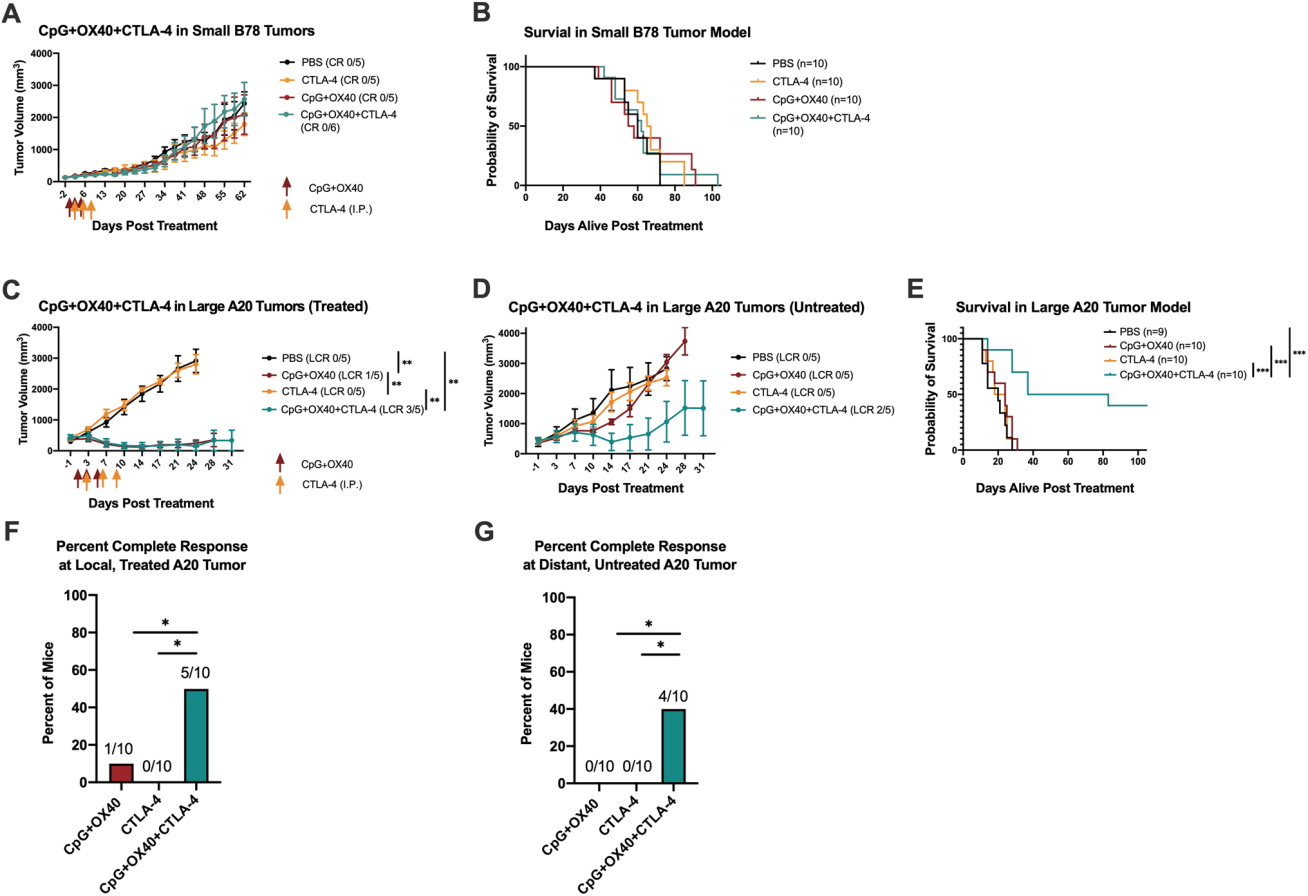
Anti-CTLA-4 enhances the anti-tumor response of CpG + OX40 in the poorly responsive A20 but not B78 tumor models. **a** Average tumor volume (± SEM) from a representative experiment and **b** combined overall survival from two independent experiments in *C57BL/6* mice bearing a single small (~ 100 mm^3^) B78 flank tumor treated with PBS (black), anti-CTLA-4 (yellow), CpG + OX40 (red), or CpG + OX40 + anti-CTLA-4 (teal). **c** Average tumor volume (± SEM) plot of the local, treated tumor and **d** distant, untreated tumor from a representative experiment, and **e** combined overall survival from two independent experiments of *Balb/c* mice treated with PBS (black), anti-CTLA-4 (yellow), CpG + OX40 (red), or CpG + OX40 + anti-CTLA-4 (teal) in mice bearing two separate large (~ 350 mm^3^) A20 tumors. [Note in Fig. 6C, the data for CpG + OX40 + anti-CTLA-4 (teal) are superimposed on the data for CpG + OX40 (red), making it hard to view]. The number of mice demonstrating a local complete response (LCR) at the treated tumor or distant untreated tumor are shown in parentheses in **(a, c)** and **(d)**, respectively. Percent of *Balb/c* mice treated with CpG + OX40 (red), anti-CTLA-4 (yellow), or CpG + OX40 + anti-CTLA-4 (teal) cured of their local, treated A20 tumor **(f)** and distant, untreated A20 tumor **(g).** *P* values for tumor volume plots were calculated using time-weighted average analysis. *P* values for overall survival were calculated via log rank test. P values for CR comparison calculated via chi-square tests. *P ≤ 0.05; **P ≤ 0.01; ***, *P* ≤ 0.001. All comparisons that are not shown with an *, **, or *** are not significantly different from each other

In the small B78 model, the addition of anti-CTLA-4 to CpG + OX40 had no effect on slowing tumor progression or overall survival (Fig. 6a, b). In the large two-tumor A20 model, the addition of anti-CTLA-4 to CpG + OX40 caused significant local tumor regression compared to PBS and anti-CTLA-4 alone and caused regression of some tumors at the distant untreated site (LCR 5 of 10 at the treated tumor and 4 of 10 at the distant tumor) (Fig. 6c, d, f, g). CpG + OX40 + anti-CTLA-4 significantly improved survival over all other groups in these studies; 40% of mice treated with CpG + OX40 + anti-CTLA-4 were cured, while all mice in all other groups died before day 40 (Fig. 6e).

## Discussion

Herein, we demonstrate tumor volume is a critical variable for CpG + OX40 sensitivity in both the B78 and A20 tumor models. While first-palpable (~ 15 mm^3^) B78 tumors respond well to CpG + OX40, 100 mm^3^ B78 tumors do not; in contrast, single 200 or 350 mm^3^ A20 tumors respond well to CpG + OX40 while single 1000 mm^3^ A20 tumors partially respond before progressing. Similar relationships between tumor volume and systemic response to CpG + OX40 are demonstrated in our two-tumor A20 model. While the preclinical tumor volumes presented in this study do not directly correlate to patient clinical tumor sizes, we believe the general relationship between anti-tumor effect and tumor size shown in this study correlates with some published clinical observations [1–5, 8]. Our preclinical results support the retrospective studies that have examined the relationship of patient tumor burden and response to immune checkpoint blockade in melanoma and non-small cell lung cancer [1–3]. Additionally, the data highlight the importance of considering tumor size when immunotherapy treatment is initiated in preclinical models. Most curative preclinical immunotherapy regimens are tested when tumors are less than 200 mm^3^ and/or have been growing for less than 11 days in the host [33]. The data examined in the context of time in the host before treatment aligns well with these generalized conclusions. Specially, B78 tumors growing in the host were significantly more likely to respond to CpG + OX40 if they had been growing for 10 days (first palpable, 15 mm^3^) rather than 21 days (small, 100 mm^3^) (Fig. 1e). In the A20 model, tumors growing in their host for 24 days (advanced, ~ 1,000 mm^3^) were less responsive than those growing for 14 days (moderate, ~ 200 mm^3^) (Fig. 2e). Our data suggest that more time in the host allows for more tumor growth and more immunoediting (potentially of the tumor cells or of the tumor microenvironment), and thus leads to more difficult to treat tumors (e.g. decreased MHC-I, MHC-II, and CD80/86 expression in large A20 tumors shown here) (Fig. 3a–c). However, this manuscript does not specifically address how the antitumor effect of CpG + OX40 is influenced by the time since implantation vs. tumor size at the time treatment is initiated. In prior work we have shown that the potency of the response of B78 tumors to treatment with intratumoral hu14.18-IL2 immunocytokine following local radiotherapy was the same for tumors of the same size, even when the time to treatment was somewhat different [34].

When syngeneic tumor cells are injected into mice, acute inflammation and necrosis occur at the injection site. This is followed by cross-presentation and antigen priming, potentially resulting in some baseline anti-tumor immune response [33]. As syngeneic tumors progress, the baseline anti-tumor immune response dampens due to immunoediting and tumor immune suppression [6, 8]. As a result, an immunotherapy regimen could be inaccurately deemed a preclinical success or failure based on when immunotherapy is initiated, how large tumors are at the start of treatment, and what preclinical model is used. In addition to the extent of tumor burden, local and systemic immunologic changes that occur with tumor progression can suppress the anti-tumor effects of immunotherapy [35, 36]. Here, we show A20 tumors decrease MHC class I and II, CD80, and CD86 expression with progression. While most clinical trials of immunotherapy regimens are based on preclinical success, many of these clinical trials do not replicate the successful preclinical anti-tumor effects. Given the financial and human costs associated with the development and performance of these clinical trials [37–39], it may be helpful to confirm preclinical regimens demonstrate efficacy across multiple tumor models, and in tumors of various sizes, when considering whether to translate these regimens to clinical testing.

We have previously published different in vivo responses to CpG between the B78 and A20 models [25]. Specifically, CpG increases OX40 expression on CD8 + T cells and CD4 + non-Tregs in the TME of A20 tumors but not B78 tumors [25]. Our data presented in this report suggest CpG is working in the B78 model to activate tumor infiltrating macrophages, which agrees with previous publications that indicate CpG activates anti-tumor macrophages via IFN-dependent pathways [40]. In the A20 model, CpG has direct anti-proliferative effects against A20 tumor cells and alters A20 tumor phenotype both in vitro and in vivo. OX40 antibody appears to function in both A20 and B78 models, in part, by decreasing tumor Treg frequency [16]. Therefore, the difference in CpG activity between the B78 and A20 models might contribute to the relative volume differences seen in the CpG + OX40 response between these two models. Clearly other factors are likely involved in this differential response, possibly including the immune capabilities of *C57BL/6* and *Balb/c* mice and the higher immunogenicity of A20 lymphoma compared to B78 melanoma.

Investigation into the role of myeloid derived suppressive cells (MDSCs), not only in the context of tumor progression, but also the response to CpG + Ox40 in-situ vaccination is an area of future interest. Some studies have demonstrated that increased levels of MDSCs negatively correlate with outcomes in patients diagnosed with cancer, including melanoma and lymphoma [41–43]. Preclinical studies have shown that MDSC accumulation in the TME significantly increases as tumor volumes increase [6]. The effects of CpG on MDSCs have been characterized in mice bearing two types of gastrointestinal tumors (C26 and CEA424-TAg models) [44]. In these experiments, CpG was shown to decrease the suppressive potential of systemic MDSCs against T cells, and this mechanism was dependent on IFN-α. While MDSCs were not examined in this manuscript, we suggest that future studies evaluate whether systemic, or tumor associated, MDSC frequencies are higher in model conditions that are less responsive to the CpG + OX40 in-situ vaccine, and whether IT injections of CpG + OX40 may act to decrease the suppressive potential of MDSCs allowing a measurable anti-tumor immune response under some model conditions.

In the A20 model, CpG + OX40 effectively cured mice bearing a single large (~ 350 mm^3^) flank tumor. If, however, a mouse had a second, similarly sized, untreated tumor on the opposite flank, the ability of CpG + OX40 to cure the local ~ 350 mm^3^ treated tumor was reduced (Fig. 2d, E vs Fig. 4g,i). Concomitant immune tolerance (CIT) is a possible explanation for the reduced local efficacy. CIT is a phenomenon describing tumor specific inhibition of *in-situ* vaccination by distant untreated tumor sites [32]. We have previously observed CIT in the two-tumor B78 model in the context of treatment with RT and IT-IC [32]. Those studies suggest that after Treg depletion with RT to the local tumor, Tregs from the distant untreated tumor repopulate these treated tumors, inhibiting the response to immunotherapy [32]. Others have published data in the two-tumor A20 model demonstrating that Tregs can migrate from one tumor to another [16]. It is possible Tregs from large, untreated A20 tumors are migrating to the treated tumor, reducing the ability of CpG + OX40 to activate a tumor-curing immune response.

In mice bearing ~ 100 mm^3^ B78 tumors, the addition of anti-CTLA-4 causes a decrease in Tregs and an increase in T cell activation [32]; even so, the addition of anti-CTLA-4 failed to improve the anti-tumor response of CpG + OX40. We have previously reported that additional T cell activation in the form of increasing OX40 dose fails to augment the anti-tumor response in the B78 model [25]. These results suggest Treg immune suppression and inadequate T cell activation are not responsible for the failed effect of CpG + OX40 in the ~ 100 mm^3^ B78 model. Instead, we hypothesize CpG + OX40 is failing to initiate a strong anti-tumor immune reaction, and thus additional immune stimulation is required. We have previously demonstrated RT can provide this additional immune activation to CpG + OX40 in ~ 100–150 mm^3^ B78 tumors by increasing IFN gene expression in the TME, increasing OX40 expression on tumor infiltrating CD4 + T cells, and increasing IFN-γ expression in CD8 + and CD4 + T cells in the tumor draining lymph node (TDLN) and spleen [25]. Conversely, additional Treg depletion via anti-CTLA-4 in the A20 model enhances both the local and systemic anti-tumor effects of CpG + OX40. This agrees with previous work on this *in-situ* vaccine in the A20 model [16]. However, our data demonstrating decreased MHC-I, MHC-II, CD80, and CD86 expression as A20 tumors progress provide additional context to the mechanism of this enhanced anti-tumor response. CD80 and CD86 are T cell co-stimulatory molecules on APCs that interact with CD28 or CTLA-4 on T cells, providing either a positive (CD28 binding) or attenuated (CTLA-4 binding) costimulatory signal to the T cell when a T cell receptor engages MHC on APCs. This axis is typically examined in the context of secondary lymphoid organs during T cell priming [31, 45]. Emerging evidence suggest this axis (e.g., CD80/86, CD28/CTLA-4) is also important outside the context of initial T cell priming and activation, demonstrating that CD4 + effector cells require sustained CD28 signaling to generate a proper type I helper response [46]. It is possible downregulated CD80 and CD86 expression in large A20 tumors is preventing the sustained CD28 signaling required for an effective adaptive anti-tumor response. Whether additional injections of CpG + OX40 (e.g. 4 or more) would overcome inhibitory effects in non-responsive models, potentially through additional CD80/86 expression changes, or increased antigen presentation, is an area of future interest. In a separate study, 9 injections of an in-situ vaccine, consisting of a STING agonist and an anti-GITR antibody, failed to improve the anti-tumor response in a two-tumor A20 model over 3 injections of the in-situ vaccine [47].

As immunotherapy continues to become part of standard of care, it will be important to better understand how tumor type and tumor burden impact the efficacy and mechanism of immunotherapy treatments. Tumor size is a criterion for the American Joint Committee on Cancer tumor staging in many cancer types, influencing treatment decisions with traditional cancer treatment modalities (i.e., RT, chemotherapy, and surgery). Emerging clinical and preclinical data, including this report, indicate tumor size and number of tumors may need to be considered when prescribing immunotherapy regimens [1–3, 7, 14, 27].

## Conclusion

The preclinical data presented here demonstrate the negative impact larger tumor burdens have on the efficacy and mechanism of CpG + OX40 *in-situ* vaccine in two separate tumor models. For comparably sized tumors, B78 melanoma was less responsive to CpG + OX40 than A20 lymphoma, and differences of these tumors’ response to CpG may, in part, account for these differences to the treatment. Anti-CTLA-4 antibody, although not effective by itself against larger tumors, can restore the anti-tumor efficacy of CpG + OX40 in larger A20 lymphomas but not in larger B78 melanomas. Our findings suggest parameters such as tumor type and tumor volume should be carefully considered when designing and testing immunotherapy regimens in preclinical settings to better predict potential clinical activity.

## Supplementary Information

Below is the link to the electronic supplementary material.

Supplementary file1 (TIFF 73517 KB)

Supplementary file2 (TIFF 72422 KB)

Supplementary file3 (TIFF 45260 KB)

Supplementary file4 (TIFF 86932 KB)

Supplementary file5 (TIFF 20040 KB)

Supplementary file6 (DOCX 17 KB)

Supplementary file7 (DOCX 15 KB)

## Data Availability

For information regarding the data presented in this work, please contact the Corresponding Author at pmsondel@humonc.wisc.edu.
