# Supplementary figures and images for: Factors impacting the efficacy of the in-situ vaccine with CpG and OX40 agonist
Source: Cancer Immunol Immunother. 2023 Apr 5;72(7):2459–71. doi: 10.1007/s00262-023-03433-3 (PMC10264285; doi:10.1007/s00262-023-03433-3)

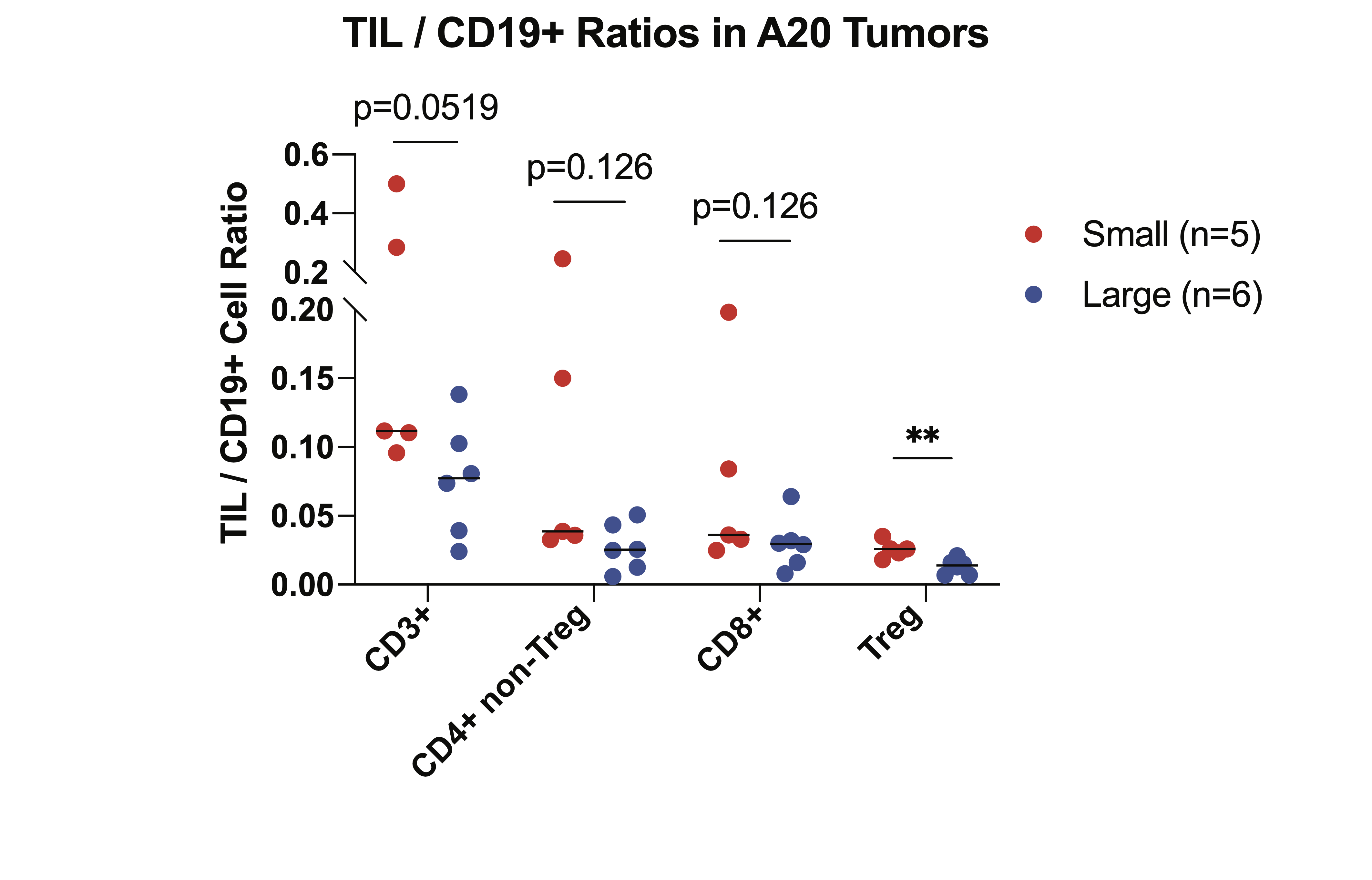

Supplement: Supplementary file 3 — Supplementary file3 (TIFF 45260 KB) [file 262_2023_3433_MOESM3_ESM.tiff]

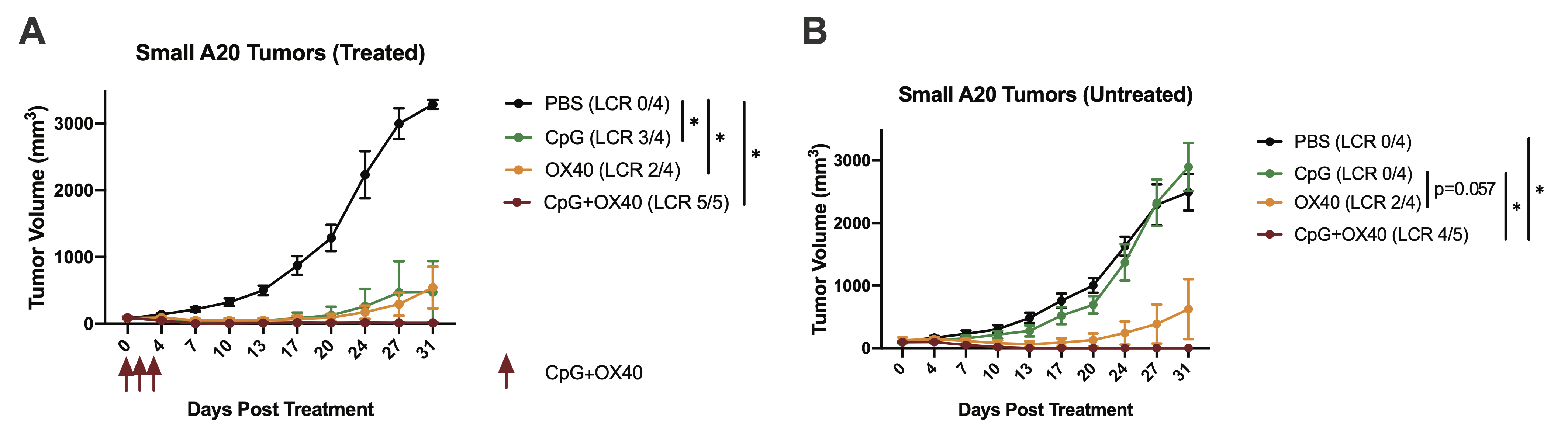

Supplement: Supplementary file 5 — Supplementary file5 (TIFF 20040 KB) [file 262_2023_3433_MOESM5_ESM.tiff]
