# Supplementary material for: Factors impacting the efficacy of the in-situ vaccine with CpG and OX40 agonist
Source: Cancer Immunol Immunother. 2023 Apr 5;72(7):2459–71. doi: 10.1007/s00262-023-03433-3 (PMC10264285; doi:10.1007/s00262-023-03433-3)
Supplement: Supplementary file 6 — Supplementary file6 (DOCX 17 KB) [file 262_2023_3433_MOESM6_ESM.docx]

**Fig. S1 *In Vivo* TIL and Tumor Phenotype Changes with B78 Tumor Progression and CpG+OX40 *In Vivo* Effect on Tumor Tregs in Small (~100 mm^3^) B78 Tumors**

**(A)** Immune cell (CD3+, CD4+ non-Treg, CD8+, Treg) to live cell ratios in untreated small (~100 mm^3^, in red) and large (~350 mm^3^, in blue) B78 tumors. The average Treg/CD3 ratio here for small tumors is 0.024 and for large tumors is 0.021.

**(B)** PD-1 MFI on CD45+ cells in the TME of small (red) and large (blue) untreated B78 tumors.

**(C)** MHC-I and MHC-II MFI on GD2+ tumor cells in the TME of small (red) and large (blue) untreated B78 tumors.

**(D)** Ratio of live GD2+ cells to total events collected in small (red) and large (blue) untreated B78 tumors.

**(E)** Ratios of Treg (CD45+ CD4+ CD25+ FoxP3+) to CD45+ cells in the TME of small B78 tumors (~100-150 mm^3^) on day 9 after IT treatment on d. 0, 2, and 4 with PBS (black) or CpG+OX40 (red). Each symbol represents the cell ratio of one mouse, and the black bar represents the group median. P values calculated via Mann-Whitney t-test. *, P≤0.05; **, P≤0.01

**Fig. S2 Flow Gating Strategy for Tumor Phenotype Analysis**

Flow cytometry gating strategy used to analyze CD19+ phenotypic MHC-I, MHC-II, CD80, and CD86 expression in small (~150 mm^3^) untreated, large (~500 mm^3^) untreated, and large (~500 mm^3^) CpG treated A20 tumors, for the data shown in Figure 3.

**Fig. S3 TIL Frequency Between Small and Large A20 Tumors**

TIL to CD19+ cell ratios (for CD3+, CD4+ non-Tregs, CD8+ and Treg cells) in small (~150 mm^3^, in red) and large (~500 mm^3^, in blue) untreated A20 tumors. The CD19 positivity was used as an indicator of the A20 tumor cells.

**Fig. S4 *In Vitro* B78 and A20 TLR-9 Expression and Macrophage Co-Culture Experiment**

**(A)** B78 or **(C)** A20 tumor cells were co-cultured with media alone (blue), 5 μg/mL CpG (red), or 50 μg/mL CpG (green) either in the absence or presence of peritoneal derived macrophages (Mφ) from *C57BL/6* (for B78) or *Balb/c* (for A20) mice. Cell proliferation (indicative of tumor cell proliferation) of each well was assessed via β-scintillation counting of [^3^H]thymidine incorporation. The data plotted here are the results from two independent experiments (n=3 per condition per experiment). Average [^3^H]thymidine uptake of each media alone condition (i.e. media without Mφ, media with *C57BL/6* Mφ, media with *Balb/c* Mφ) was calculated for each separate experiment, then used to determine intra-experimental fold-change differences for each sample in that particular macrophage condition. Each symbol represents the fold change from one well. (B) B78 or (D) A20 tumor cells were harvested, fixed, permeabilized, and stained with anti-TLR-9 (blue line) or isotype control (gray shaded) to evaluate for TLR-9 expression. (E) A20 cells were co-cultured with media alone (black) or 3 μg/mL of CpG (green) for 72 hours before A20 cells were harvested and analyzed via flow cytometry. Average (+/- SEM) median fluorescence intensity (MFI) for CD80, CD86, MHC-I, and MHC-II on A20 tumor cells cultured without (black) or with CpG (green). P values calculated via two-way ANOVA with Tukey’s multiple comparison correction. *, P≤0.05; **, P≤0.01; ***, P≤0.001; ns – no significance

**Fig. S5 Validation of the Local and Distant Anti-Tumor Effects of CpG Alone and OX40 Alone in the Two-Tumor A20 Model**

*Balb/c* mice bearing two separate small (~100 mm^3^) A20 tumors were treated with PBS (black), CpG (green), OX40 (orange), or CpG+OX40 (red). Average tumor volume (+/- SEM) are shown for the treated tumor **(A)** and the distant untreated tumor **(B)**. The number of mice demonstrating a local complete response (LCR) at the treated tumor or distant untreated tumor are shown in parentheses in **(A)** and **(B)**, respectively. P values for tumor volume plots were calculated using time-weighted average analysis. *, P≤0.05. All comparisons that are not shown with an * are not significantly different from each other.
