# Supplementary material for: Factors impacting the efficacy of the in-situ vaccine with CpG and OX40 agonist
Source: Cancer Immunol Immunother. 2023 Apr 5;72(7):2459–71. doi: 10.1007/s00262-023-03433-3 (PMC10264285; doi:10.1007/s00262-023-03433-3)
Supplement: Supplementary file 7 — Supplementary file7 (DOCX 15 KB) [file 262_2023_3433_MOESM7_ESM.docx]

**Table S1**

| **Target** | **Fluorophore** | **Clone** | **Distributor** |
| --- | --- | --- | --- |
| CD3 | PE-Cy5 | 145-2C11 | BioLegend |
| CD4 | BV785 | GK1.5 | BioLegend |
| CD8 | APC-R700 | 53-6.7 | eBioscience |
| CD16/32 | N/A | 93 | BioLegend |
| CD19 | PE-Cy5 | 6D5 | BioLegend |
| CD19 | APC | 6D5 | BioLegend |
| CD19 | FITC | 6D5 | BioLegend |
| CD25 | BB515 | PC61 | eBioscience |
| CD45 | BV510 | 30-F11 | BioLegend |
| CD80 | PE-Cy5 | 16-10A1 | BioLegend |
| CD86 | PE-Cy7 | BU63 | BioLegend |
| GD2 | PE | 14G2a | BioLegend |
| PD-1 | BV421 | EH12.2H7 | BioLegend |
| OX40 | PE | OX-86 | BioLegend |
| CTLA-4 | APC | UC10-4B9 | eBioscience |
| FoxP3 | PE-Cy7 | FJK-16s | eBioscience |
| MHC-I | PE | Cat. 553580 | Pharmingen |
| MHC-II | BV605 | M5/114.15 | BioLegend |
| TLR-9 | PE | S18025A | BioLegend |
| Rat IgG | PE |  | BioLegend |
| Dead cells | GR510 | N/A | Tonbo Biosciences |
| Dead cells | GR780 | N/A | Tonbo Biosciences |

Table S1 - Antibodies used in flow cytometry experiments
